# Atomistic insights into the morphological dynamics of gold and platinum nanoparticles: MD simulations in vacuum and aqueous media

**DOI:** 10.3762/bjnano.15.81

**Published:** 2024-08-07

**Authors:** Evangelos Voyiatzis, Eugenia Valsami-Jones, Antreas Afantitis

**Affiliations:** 1 NovaMechanics Ltd., Nicosia 1070, Cyprus; 2 School of Geography, Earth and Environmental Sciences, University of Birmingham, Birmingham B15 2TT, United Kingdom

**Keywords:** crystallization, gold, molecular dynamics, nanoparticles, platinum

## Abstract

The thermal response of gold and platinum spherical nanoparticles (NPs) upon cooling is studied through atomistic molecular dynamics simulations. The goal is to identify the morphological transformations occurring in the nanomaterials as well as to quantify their dependence on temperature, chemistry, and NP size. For diameters smaller than 3 nm, the transition temperature from a melted/amorphous to a highly crystalline state varies considerably with NP size. For larger NPs, the transition temperature is almost diameter-independent, yet it differs considerably from the transition temperature of the respective bulk materials. The platinum NPs possess a higher level of crystallinity than the gold counterparts under the same conditions because of the stronger cohesive forces that drive the crystallization process. This observation is also supported by the simulated X-ray powder diffraction patterns of the nanomaterials. The larger NPs have a multifaceted crystal surface, and their shape remains almost constant regardless of temperature variations. The smaller NPs have a smoother and more spherical surface, and their shape varies greatly with temperature. By studying the variation of nano-descriptors commonly employed in QSAR models, a qualitative picture of the NPs’ toxicity and reactivity emerges: Small/hot NPs are likely more toxic than their large/cold counterparts. Because of the small size of the NPs considered, the observed structural modifications are challenging to be studied by experimental techniques. The present approach can be readily employed to study other metallic and metal oxide nanomaterials.

## Introduction

Nanomaterials, that is, materials with dimensions in the range of 1–100 nm [1–2], are central to a variety of developments in science and technology, from medicine and engineering to the environment and energy. Because of their small size, nanoparticles (NPs) have only been discovered relatively recently, although they have been present in the environment throughout earth’s and human history, emerging from various sources including biological, anthropogenic, and geological processes [3]. Only a few decades ago, NPs attracted attention because of their size-dependent chemical and physical properties [4]; nowadays, they are commercially available and exploited in several sectors such as optics, automotive, electronics, and healthcare [5–6]. A notable category of engineered NPs is comprised of metal and metal oxide NPs, which rank among the highest in production volume. They have already found widespread applications in technological advancements such as photovoltaics, catalysis, gas sensors, fuel cells, and adsorbents [7–8]. This prevalence is attributable to their distinctive properties, including superparamagnetism, piezoelectricity, certain optical characteristics [9–13], and the enormously high surface-to-volume ratio. These special properties derive from their small size, rather than their chemical composition. Given the broad spectrum of possible applications, NPs have the potential to profoundly influence society [14].

Despite the numerous studies and advances [15–20], the rational design of NPs, especially the prediction of their structural modifications in industrial processes, such as rapid heating or cooling, is still hindered by several factors. For instance, observing NPs under real working conditions remains a challenge for experimentalists, as the capability to conduct in situ experiments has not yet been fully realized [21]. Experimental methods, such as confocal microscopy [22], laser light scattering [23], and optical microscopy [24], have provided accurate estimates of nucleation rates and critical nucleation sizes, but little data have been produced for the sub-micrometer size regime regarding crystal facet formation and the mechanism of crystal growth. Moreover, a fundamental prerequisite for NPs is the consistency in their shape, surface characteristics, and crystallinity. Nevertheless, developing straightforward and widely applicable approaches to crystallize or melt NPs uniformly, with precise control, remains a significant challenge [25]. For instance, it has been shown that atomic stresses at the NP surface are crucial in phase transitions below a certain critical NP size [26]. Although it is understood that, qualitatively, the surface stress generates an effect comparable to an externally applied compressive pressure on the NP, a quantitative description is missing. While there have been some promising theoretical models [27] and in situ observations [28], crucial elements that can harmonize thermodynamic and kinetic controls remain unclear at the nanoscale.

The plentiful theoretical efforts to understand and interpret structural modifications in metals upon thermal treatment can be traced back to the seminal works of Lindemann [29] and Pawlow [30]. Recent developments and the current state of the art have been summarized in the reviews of Mei and Lu [31] and Alcoutlabi and McKenna [32]. Emphasis has been placed on relating the melting temperature of a NP to its size by adapting theories suitable for bulk materials to NPs; examples include the classical nucleation theory [33], phenomenological models [34–36], as well as molecular simulations [37–40]. A molecular dynamics (MD) study of shape transformation and melting of tetrahexahedral Pt NPs has been carried out by Wen et al. [41]. Wang et al. employed ab initio MD to describe the melting of icosahedral Au nanoclusters [42]. The structural and thermal stability of high-index-faceted Pt NPs was addressed by Zeng et al. [43]. Similarly, the thermal stability of unsupported Au NPs was investigated by molecular dynamics [44]. The strong decrease of the melting point of small Au NPs compared to bulk Au was quantified by Qiao et al. [45]. Nayebi and Zaminpayma [46] as well as Shim et al. [47] studied the crystallization of liquid Au NPs. The dependence of the surface energy of gold NPs on their size and shape was looked into by Holec et al. [48], while Martin et al. considered silver NPs [49]. A comparative study of surface disorder in Au and Ag NPs upon cooling was carried out by Agudelo-Giraldo et al. [50]. Chushak and Bartell considered the structural modifications upon freezing of several molten Au clusters consisting of 1157 atoms [51]. Some light on the microscopic origin of the anisotropic growth of gold NPs has been cast via molecular dynamics simulations [52]. In a similar way, Lümmen and Kraska investigated the homogeneous nucleation and cluster growth of Pt clusters from supersaturated vapour [53]. A combined molecular dynamics and X-ray diffraction analysis of gold NPs has been carried out by Kamiński et al. [54]. The dynamical stability and vibrational properties of Pt nanoclusters by ab initio methods were investigated by Maldonado et al. [55]. A comprehensive review of Pt NPs has been compiled by Quinson and Jensen [56].

The aim of the present work is twofold, namely, (i) to discern the structural modifications in initially spherical NPs occurring upon rapid cooling and (ii) to link these modifications to the NP size, as quantified by the initial diameter, the NP chemical composition, and the temperature. To this end, atomistic molecular dynamics simulations have been performed for gold (Au) and platinum (Pt) NPs with diameters from 1 to 8 nm for a range of temperatures. Bulk Au and Pt materials share the same unit cell of the crystal structure, yet they differ in the strength of their energy interactions. The morphological changes in the NPs are measured using both atomic parameters, such as the coordination number and the Berry parameter, and cluster parameters, such as the X-ray powder diffraction pattern and the asphericity parameter. Furthermore, we extract qualitative information regarding the toxicity and reactivity of these NPs by monitoring the behaviour of nano-descriptors commonly employed in quantitative structure–activity relationship (QSAR) models and by measuring the water–NP energetic interactions. The extracted information from our simulations complements experimental techniques by providing insights into phenomena occurring at time and length scales that are challenging to capture experimentally.

## Methods

We performed atomistic MD simulations of spherical Au and Pt NPs in vacuum and in aqueous media. The considered NP diameters and the number of atoms in each NP are presented in Table 1. The potential energy of the NPs is described by the EAM/alloy force field; the parameters proposed by Grochola et al. [57] for the Au NPs and by O'Brien et al. [58] for the Pt NPs are adopted. For both force fields, files containing all required parameters in suitable LAMMPS format have been obtained from the NIST interatomic potentials repository (https://www.ctcms.nist.gov/potentials/) [59–60].

**Table 1:** NP diameters and number of atoms in Au and Pt NPs.

NP diameter (nm)	Number of atoms in NP	

	Au NP	Pt NP

1.0	43	32
2.0	249	257
3.0	887	846
4.0	1985	2015
5.0	3925	3918
6.0	6699	6817
7.0	10641	10791
8.0	15707	16149

The initial configurations of the Au (Pt) NPs are constructed as follows: A supercell consisting of 2048 Au (Pt) atoms is obtained by replicating the face-centered cubic (FCC) unit cell 8 × 8 × 8 times. The supercell is then simulated for 1 ns in the canonical (NVT) ensemble at 300 K. The Langevin thermostat is employed with a coupling time of 0.1 ps. A time step of 1 fs using the velocity-Verlet integration scheme is used. The system is subsequently heated to 1400 K (2100 K), that is, the melting point of bulk Au (Pt), in the isothermal-isobaric (NPT) ensemble at 101.3 kPa with a constant heating rate of 10 K/ns. The Langevin thermostat and the Nosè–Hoover barostat [61] are employed with coupling times of 0.1 and 1.0 ps, respectively. When the heating stage is completed, further equilibration is performed for 20 ns in the NPT ensemble at 101.3 kPa and 1400 K (2100 K). The final amorphous system is replicated several times along all three Cartesian coordinates so that a spherical NP with the desired diameter can be curved out.

**Figure 1:**
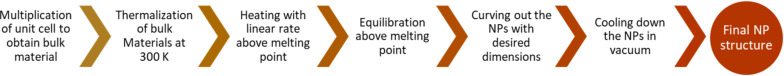
Schematic of the computational procedure utilized to generate nanoparticle configurations at various temperatures.

Afterwards, the Au (Pt) NPs are placed in vacuum, and the systems are cooled down to 100 K following the single-step procedure of Martin et al. [49]. In each step, the temperature is decreased instantaneously by 100 K, and the systems are relaxed by performing a MD simulation of 20 ns in the NVT ensemble. In total, this procedure is employed 13 (20) times for all Au (Pt) NPs until the temperature reaches 100 K. Configurations are sampled every 10 ps from the last 1 ns of each cooling step. A schematic of the computational steps to generate the NP configurations is shown in Figure 1. Although the employed procedure results in extremely high heating and cooling rates compared to the experimental ones, it has been shown to yield representative structures that are in good agreement with the ones observed via X-ray diffraction for a number of nanomaterials such as CuO NPs [62], TiO_2_ NPs [63], as well as carbon [64] and Ag [65] nanostructures.

We also simulated Au and Pt NPs in aqueous solutions at 300 K, that is, close to room temperature. The interactions among the water molecules are described by the SPC/E model [66]. The interactions among the water molecules and the Au (Pt) atoms are calculated by the force field of Merabia et al. [67] (Brunello et al. [68]). The initial configuration of a hydrated NP is obtained by placing the NP inside a pre-equilibrated water configuration and removing all water molecules that are closer than 0.5 nm from any Au (Pt) atom. The resulting system is equilibrated for 10 ns in the NPT ensemble at 101.3 kPa and 300 K. The Nosè–Hoover thermostat and barostat are employed with coupling times of 0.1 and 1.0 ps, respectively. After equilibration, a subsequent simulation for 1 ns takes place in the NPT ensemble at 101.3 kPa and 300 K where configurations are sampled every 10 ps. All simulations are performed with the LAMMPS code [69], and atomistic configurations are visualized using the Ovito software [70].

The structural modifications occurring in the NPs are identified by monitoring the temperature variation of atomic and cluster parameters. One such atomic quantity is the Berry parameter, δ, which forms a distance–fluctuation criterion to identify first-order transitions, for example, from liquid to solid phases [71–72]. It is given by


[1] $$\delta =\frac{2}{N\left(N-1\right)}\sum_{i=1}^{N-1}\sum_{j=i+1}^{N}\frac{\sqrt{{\langle r_{ij}^{2}\rangle }_{t}-{\langle r_{ij}\rangle }_{t}^{2}}}{\langle r_{ij}\rangle },$$

where *N* is the number of atoms in the NP, *r_ij_* is the distance between the *i*-th and the *j*-th atom and ⟨…⟩*_t_* denotes time averaging. A critical value close to 0.05 signifies the occurrence of a phase transition in a cluster of atoms. Additional atomic parameters are the average potential energy, force, and coordination number per atom. These quantities have also been employed as descriptors in nano-QSAR models to successfully predict the toxicity of NPs [73–75]. The average force per atom, *f*, is computed as 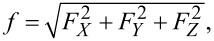 where *F_k_* is the *k*-th Cartesian component of the force vector **F**. The coordination number of an atom is defined as the number of its neighbouring atoms that lay within a given distance. For the Au (Pt) atoms, a distance of 0.32 (0.30) nm is used. Additionally, every atom is assigned to a structural type matching a known crystal form (FCC, body-centered cubic (BCC), hexagonal close-packed (HCP), icosahedral, or amorphous) based on the Ackland–Jones bond-angle method [76] as implemented in Ovito.

One of the employed cluster parameters is the surface area-to-volume ratio of a NP. The surface area is calculated by the alpha-shape method with a probe sphere radius of 0.3 nm [77] as available in Ovito [70]. The volume is determined by performing a Delaunay tessellation on the atomistic configuration and summing up the volumes of the resulting tetrahedra. The tessellation is carried out using the Qhull library [78]. The shape of a NP is quantified by the asphericity, *b*, the acylindricity, *c*, and the relative shape anisotropy, κ^2^, parameters [79]. Let 
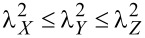
 denote the eigenvalues of the gyration tensor. The shape parameters are given by:


[2]

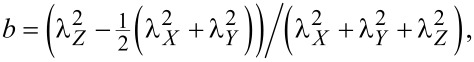




[3] $$c=\left(\lambda_{Y}^{2}-\lambda_{X}^{2}\right)/\left(\lambda_{X}^{2}+\lambda_{Y}^{2}+\lambda_{Z}^{2}\right),$$

[4] $$\kappa^{2}=\frac{3}{2}\frac{\lambda_{X}^{4}+\lambda_{Y}^{4}+\lambda_{Z}^{4}}{{\left(\lambda_{X}^{2}+\lambda_{Y}^{2}+\lambda_{Z}^{2}\right)}^{2}}-\frac{1}{2}.$$

Complementary information regarding the NP morphology is obtained from simulated X-ray powder diffraction patterns as determined by Debye functional analysis [80]. The intensity of the diffracted coherent radiation, *I*, is given by


[5] $$I=\sum_{i=1}^{N-1}\sum_{j=i+1}^{N}g_{i}\left(\beta \right)g_{j}\left(\beta \right)\sin\left(2\pi \beta r_{ij}\right)/\left(2\pi \beta r_{ij}\right),$$

where β = 2sin(θ)/λ, λ is the wavelength of the incident radiation, and 2θ is the scattering angle. The scattering functions *g* are computed using the expressions proposed by Cromer and Mann [81]. A λ value of 0.15418 nm is employed, representing Cu Kα radiation. Python codes to compute the Berry parameter and the X-ray powder diffraction pattern of a NP are available at https://github.com/evoyiatzis/Jupyter-Notebooks.

## Results and Discussion

**Figure 2:**
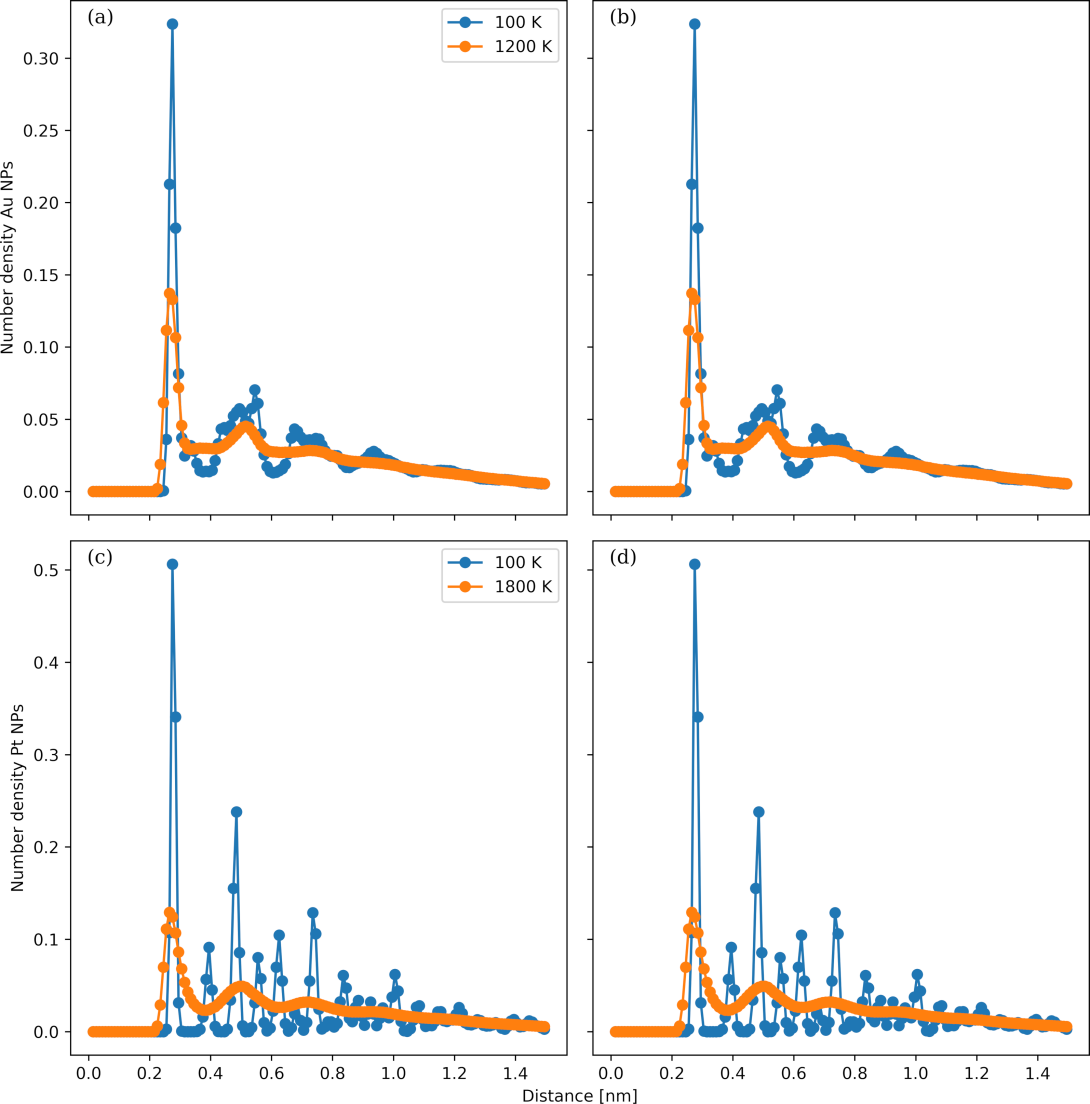
Radial number density in Au (panels a and b) and Pt (panels c and d) NPs as a function of the distance measured relative to a chosen reference atom. The NP diameters considered are 2 nm (panels a and c) and 8 nm (panels b and d). The temperatures of the Au NPs are 100 K (blue line) and 1200 K (orange line). The temperatures of the Pt NPs are 100 K (blue line) and 1800 K (orange line).

The radial number density distributions in selected Au and Pt NPs for two temperatures are shown in Figure 2. The NP diameters are 2 nm (Figure 2a,c) and 8 nm (Figure 2b,d). The considered temperatures for the Au NPs (Figure 2a,b) are 100 K (blue line) and 1200 K (orange line), while, for the Pt NPs (Figure 2c,d), they are 100 K (blue line) and 1800 K (orange line). Regardless of chemical composition and NP diameter, the number density distributions at high temperatures are similar, and their shape is typical of liquid and amorphous materials. They have two pairs of peaks and valleys, which correspond to the first and second coordination shells. For the Au NPs, the peaks are located at 0.275 nm and multiples of this distance, while, for the Pt NPs, they lie at roughly 0.250 nm and its multiples. For long distances, the number density distribution reaches a plateau value, which implies that, for sufficiently large distances, the atoms are uniformly distributed in the NP. Thus, there is no persistent structural feature present in the materials. The number density distribution for the two large NPs at 100 K is characterized by sharp and well-separated peaks, which is a telltale sign of the existence of crystal domains in the NPs. The positions of the peaks in the Au NPs are located at slightly greater distances than in the Pt NPs because of the shorter dimensions of the Pt unit cell. For the small NPs at 100 K, new peaks have emerged in the number density distribution, but they are not as sharp as in the case of the large NPs. Moreover, the height of the peaks is much smaller compared to those for the large NPs. This feature reflects a lower degree of crystallinity for the small relative to the large NPs and the fact that the nanomaterials are in a supercooled amorphous, and not liquid, state.

**Figure 3:**
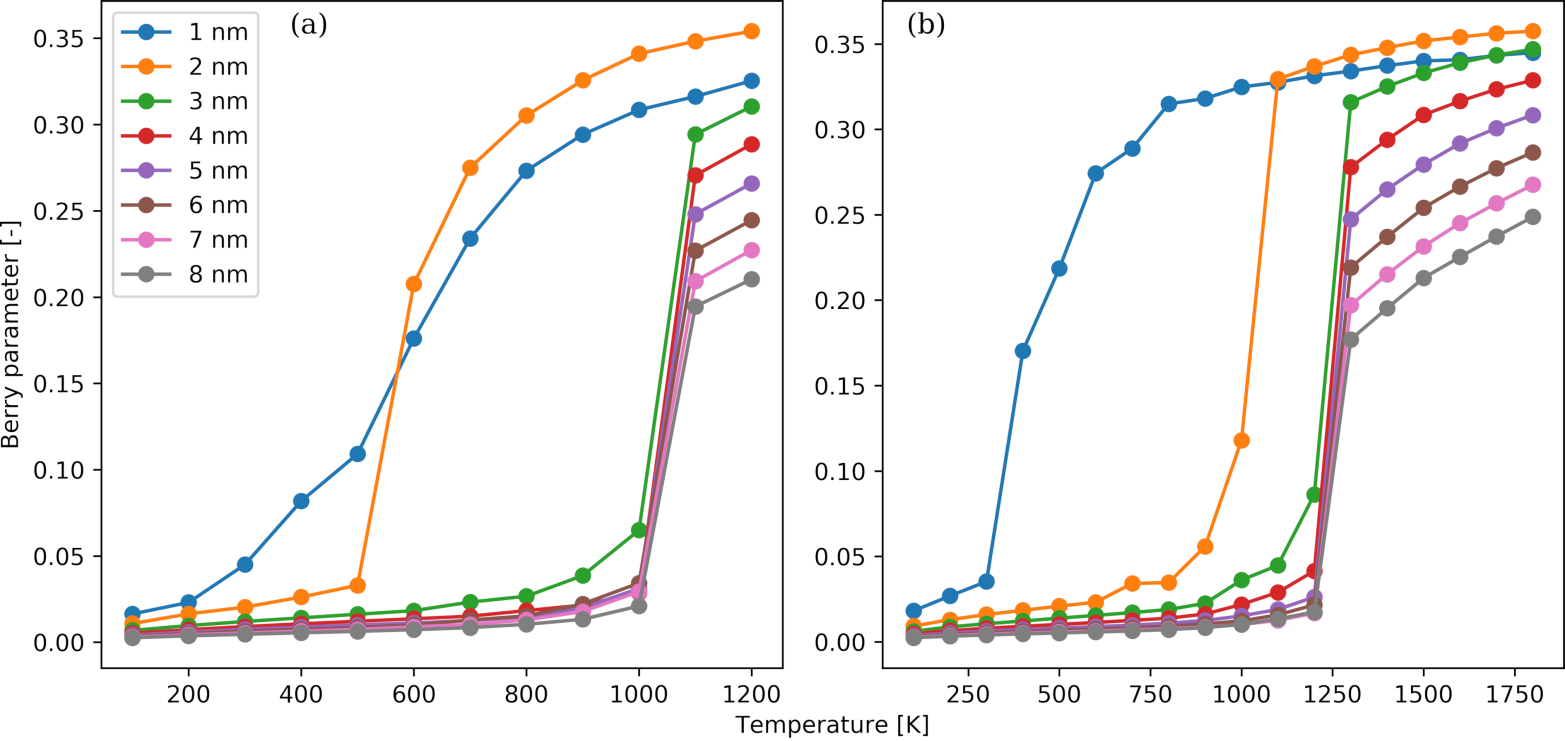
Temperature dependence of the Berry parameter of Au (panel a) and Pt (panel b) NPs. The NP diameters range from 1 to 8 nm.

The temperature dependence of the Berry parameter, δ, of the Au and Pt NPs is shown in Figure 3a and Figure 3b, respectively. The NP diameters vary from 1 to 8 nm. The Berry parameter quantifies the mobility of the atoms in the NPs by measuring the spatial fluctuations around their mean atomic position. In all cases, δ becomes larger with increasing temperatures. For the Au NPs with a diameter larger than 2 nm, a sharp drop in the δ curves takes place between 1000 and 1100 K; the δ value becomes smaller than the critical value of 0.05, and a first-order transition is identified. The temperature where the transition occurs is approximately 200 K smaller than the melting temperature of bulk crystalline Au, which is close to 1100 K. This difference stems from the higher mobility of the Au atoms in a finite-size cluster placed in vacuum compared to the atomic mobility in a dense crystal/amorphous bulk material. For the Au NP with a diameter of 2 nm, a similar steep drop takes place at even lower temperatures of 500 and 600 K. This large shift in the transition temperature indicates that the NP diameter of 2 nm is smaller than a critical size that would yield a behaviour comparable to bulk Au. For the last case of Au NPs with a diameter of 1 nm, we observe a smooth δ curve, and the critical δ value is reached at approximately 300 K. A similar behaviour is observed for the Pt NPs. For all Pt NPs with diameter larger than 2 nm, a phase transition is identified between 1200 and 1300 K. The difference between the melting temperature of bulk Pt, which is close to 2100 K, and 1200 K is much larger than the respective temperature difference in the Au case. This can be attributed to the lower cohesive energy of the Au unit cell compared to the Pt unit cell. Although both Au and Pt share the same FCC structure, the cohesive energy is larger in Pt; thus, the restoring forces to the equilibrium crystal positions are stronger. This is also supported by the findings shown below in Figure 6. The transition temperature is lowered to 900 and 300 K for the NPs with diameters of 2 and 1 nm, respectively. The δ curve becomes smooth for the NP with a diameter of 1 nm akin to the Au case.

**Figure 4:**
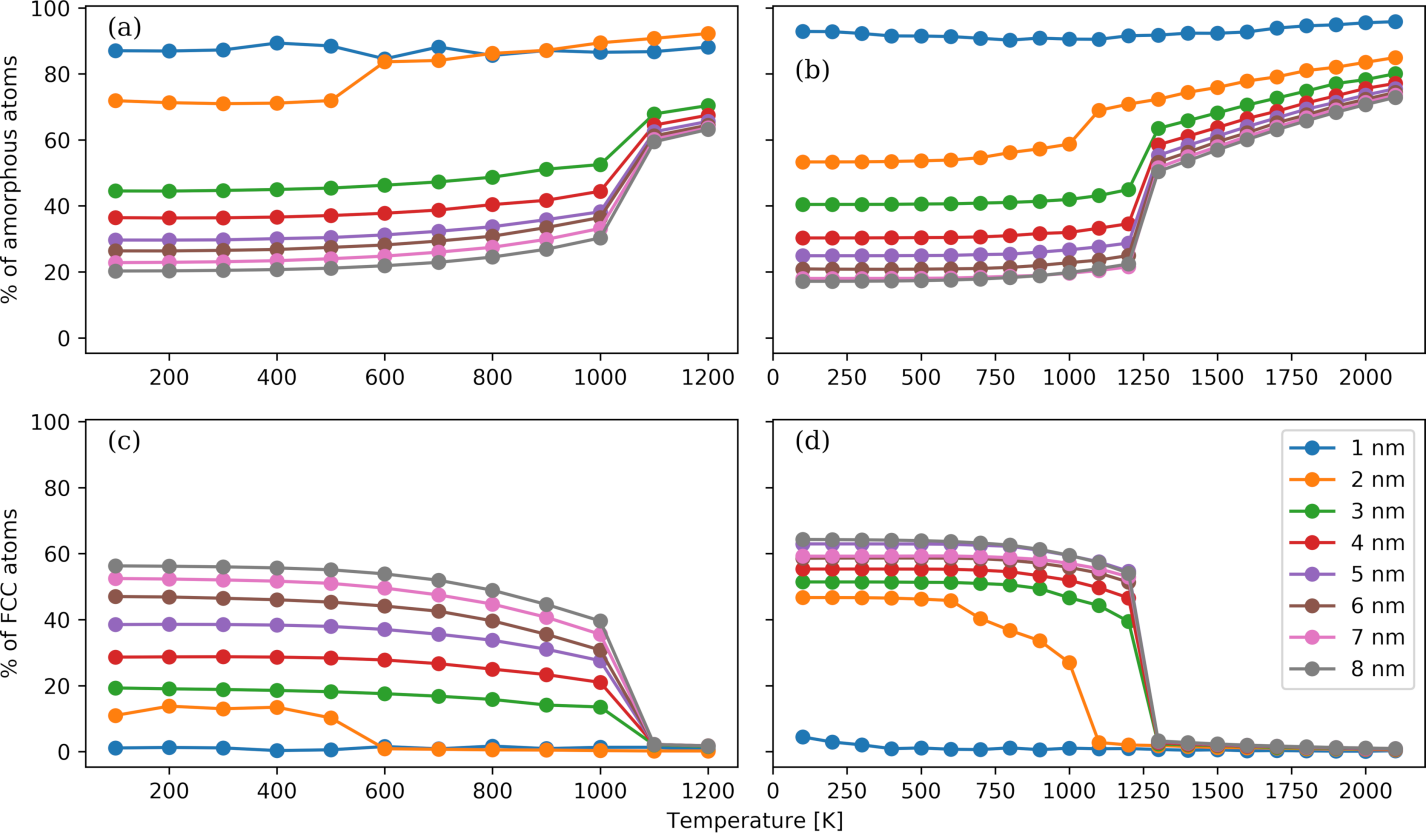
Temperature variation of the percentage of amorphous atoms (panels a and b) and FCC atoms (panels c and d) for Au (panels a and c) and Pt (panels b and d) NPs. The NP diameters range from 1 to 8 nm.

Furthermore, we utilized the Ackland–Jones method to estimate the degree of crystallinity of each NP and monitor the crystallization process (Figure 4). The temperature dependence of the percentage of identified atoms belonging to an amorphous (Figure 4a,b) and to an FCC (Figure 4c,d) domain is shown for the Au (Figure 4a,c) and Pt (Figure 4b,d) NPs. The NP diameters range from 1 to 8 nm. We note that, for both Au and Pt NPs, the sum of the two percentages is not equal to 100%. The reason is that a small proportion of the atoms are classified as atoms belonging to alternative structures, that is, BCC, HCP, or icosahedral structures. These structures should be considered as intermediate unstable states or as grain boundaries of the thermodynamically stable FCC domains in the NPs. In all cases, the percentage of FCC atoms at high temperatures is almost zero, and the amorphous atoms have the highest abundance. This observation supports the assumption that the NPs have been fully melted and there are no remnants of the initial FCC structure. With the exception of the NPs with a diameter of 1 nm, the percentage of FCC atoms exhibits a strong increase when the transition temperature is reached, which is coupled to a rapid decrease in the fraction of amorphous atoms. The transition temperature in each case is the same as the one identified by monitoring the Berry parameter. For the NPs with a diameter of 1 nm, there is a very weak dependence of both amorphous and FCC atoms on the temperature, and the fractions of FCC atoms are close to zero. This finding supports the idea that the smallest NPs are supercooled amorphous nanomaterials with no persistence of any structural features. For a given diameter, the final percentage of FCC atoms in Pt NPs is always higher than the one in Au NPs. This observation could be attributed to the higher cohesive energy of the Pt unit cell compared to the Au unit cell and the stronger interactions between Pt atoms than between Au atoms. Moreover, there is a stronger dependence of the number of FCC atoms on the NP diameter. Indirect evidence of the crystallization taking place in the NPs is provided by the visualizations shown in Figure S1 and Figure S2 of Supporting Information File 1. Snapshots of Au and Pt configurations with diameters of 2 and 8 nm are presented. A simple visual inspection confirms the formation of a multifaceted crystal surface at low temperature, while a smoother and uniform surface is seen at high temperature.

**Figure 5:**
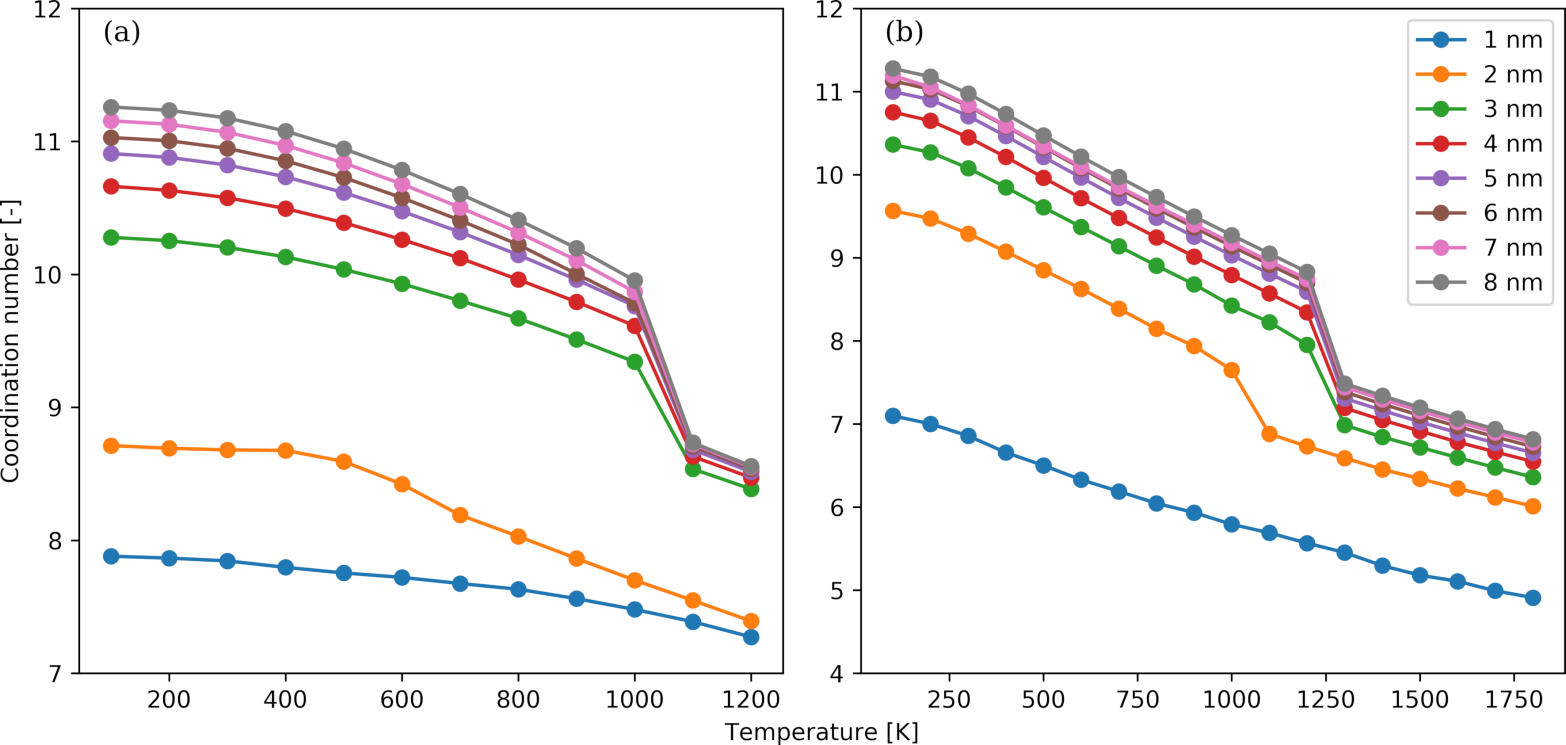
Temperature dependence of the average atomic coordination number for Au (panel a) and Pt (panel b) NPs. The NP diameters range from 1 to 8 nm.

The temperature dependence of the average coordination number as a function of the NP diameter is shown in Figure 5 for the Au (Figure 5a) and Pt (Figure 5b) NPs. We observe that an increase in temperature results in a smaller coordination number. The temperature dependence is more pronounced for the NPs with diameters larger than 2 nm. For these NPs, an abrupt reduction of the coordination number occurs close the transition temperature identified by the Berry parameter. The most stable crystal unit cell for both bulk materials under relevant conditions is the FCC structure [82] with a lattice constant of 0.4065 nm for Au and 0.3912 nm for Pt, that is, the latter being slightly shorter. The coordination number in an FCC unit cell without defects and for cutoff distances somewhat larger than the lattice constant is 12. Thus, for the lower temperatures considered, such as 100 K, the atoms exhibit preferably the equilibrium FCC structure, and the coordination number tends to the theoretical value of 12. Additionally, an increase in temperature leads to less dense NPs, as indicated by the number density variation in Figure 2, and in greater spatial fluctuations from the lattice positions dictated by the FCC structure. Moreover, the formation of crystal structures such as BCC and HCP, which have a lower density than FCC, becomes less energetically prohibitive. When focusing on the morphology of the NPs, the coexistence of several small crystal domains interconnected via amorphous grain boundaries is favoured at higher temperatures, while the crystallization process at lower temperatures leads to larger crystal domains with smaller boundaries as pointed out in Figure 4.

**Figure 6:**
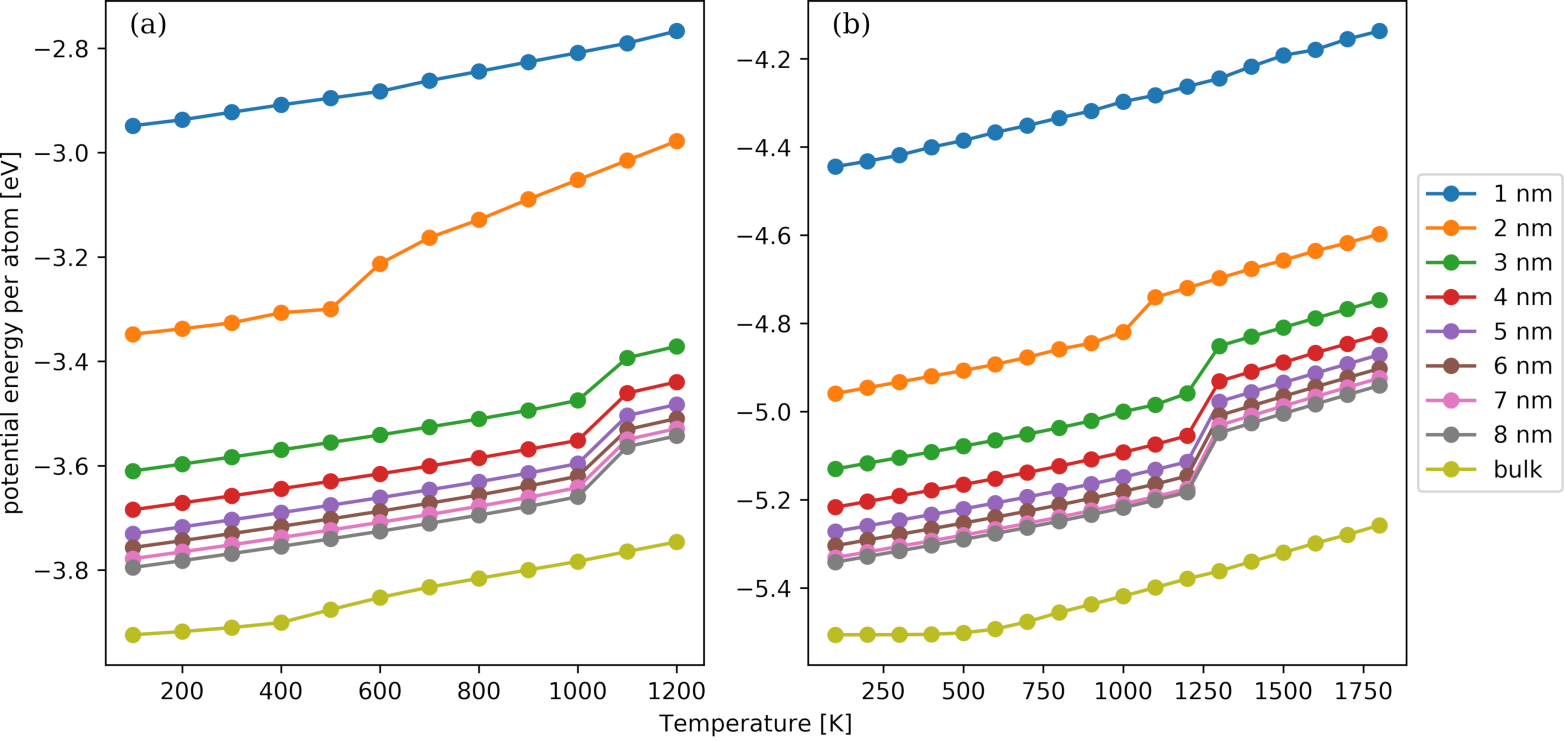
Temperature dependence of the average potential energy per atom for Au (panel a) and Pt (panel b) NPs and bulk FCC crystals. The NP diameters range from 1 to 8 nm and increase from top to bottom. The last curve corresponds to the bulk FCC crystals.

The temperature dependence of the average potential energy of an atom as a function of the NP diameter is shown in Figure 6 for the Au (Figure 6a) and Pt (Figure 6b) NPs. The NP diameters range from 1 to 8 nm. The temperature dependence of bulk FCC Au and Pt crystals is also included in the panels. We observe that the magnitude of the potential energy becomes greater with increasing NP diameter or with decreasing temperature. For the NPs with a diameter larger than 1 nm, a significant decrease in the potential energy occurs, which is another manifestation of a first-order phase transition. When comparing Au NPs with Pt NPs with the same diameter and at the same temperature, we observe that the potential energy is higher in the case of Pt. It reflects that Pt crystal structures have a higher cohesive energy than the respective Au ones [82] and that amorphous Pt materials have a greater density than Au ones. The NPs with 1 nm diameter have qualitatively the same temperature dependence as the bulk materials, that is, a proportional linear relationship can be seen. This behaviour suggests that the NPs do not undergo any phase transition in the considered temperature range; this is similar to their bulk counterparts, which display only one stable phase. The dependence of the potential energy on the NP diameter is more pronounced for the smaller NPs considered, while an almost marginal difference between the NPs with 7 and 8 nm is noted. Nevertheless, the gap between the NP with 8 nm diameter and the bulk material is large enough to suggest that finite-size effects as well as geometrical deviations from a flat surface are strong for the considered diameters.

With the knowledge of the coordination number and the potential energy per atom at hand, we can utilize our in-house QSAR model [75] to assess qualitatively the effect of temperature and NP size on toxicity. Although the model has been trained on data for Ag, TiO_2_, and CuO NPs, its applicability to Au and Pt NPs is justified since Ag, Au, and Pt are pure metallic NPs and the corresponding bulk materials crystallize in FCC structures where only the lattice spacing differs. We observe that the small NPs at high temperatures have a larger score than the larger ones at lower temperatures. The classification of the adverse effects is “high” for the former and “low” for the latter ones. The critical NP size for the classification is 4 nm for both Au and Pt NPs.

**Figure 7:**
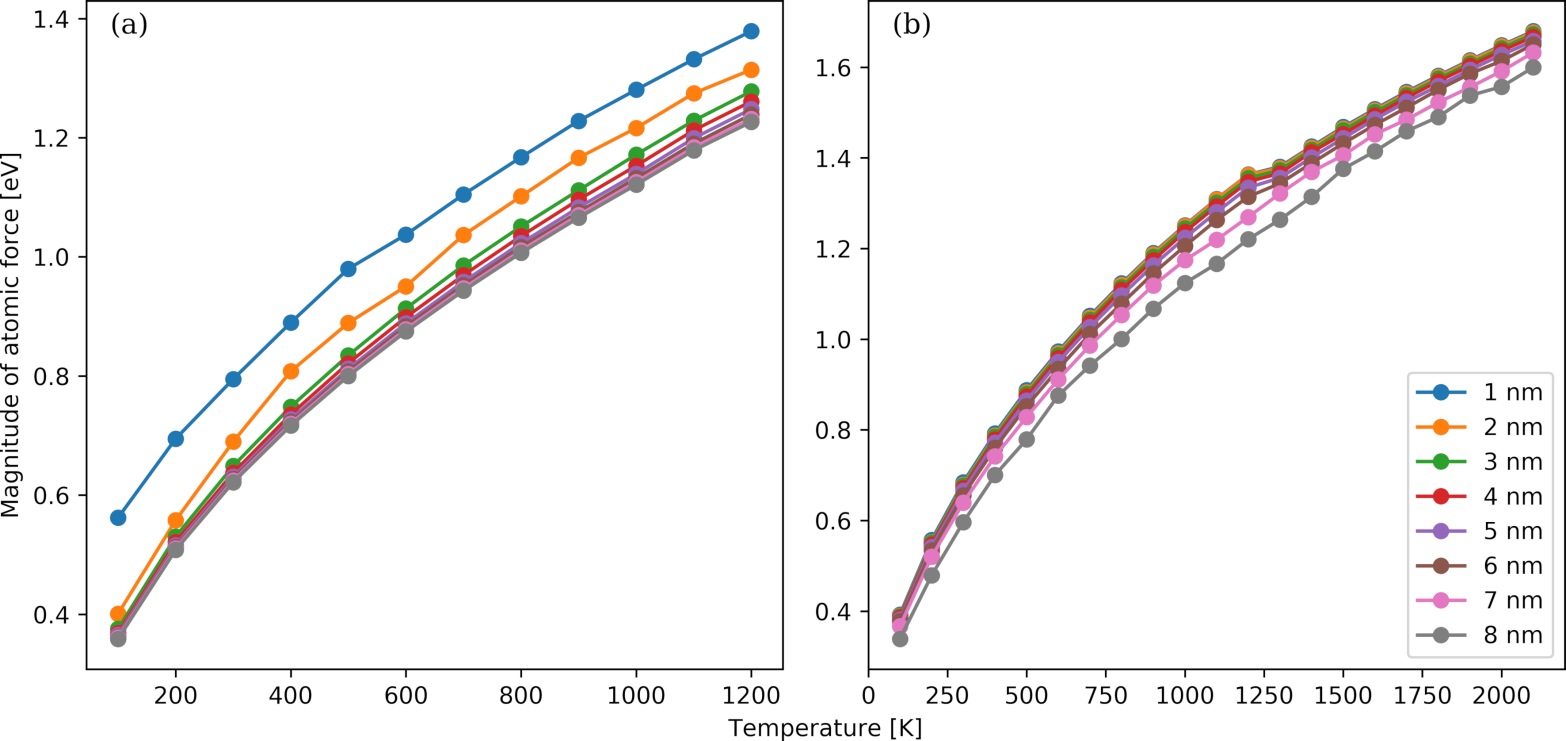
Temperature dependence of the magnitude of atomic force for Au (panel a) and Pt (panel b) NPs. The NP diameter ranges from 1 to 8 nm.

The last atomic quantity we are exploring is the mean force applied to an atom. The temperature dependence of this parameter as a function of the NP diameter is shown in Figure 7 for Au (Figure 7a) and Pt (Figure 7b) NPs. The mean force becomes greater when the temperature is raised or the NP diameter is increased. For a fixed NP diameter, a temperature reduction results in smaller spatial fluctuations, expanded in size and number of crystal zones, as well as more ordered NP configurations that are closer to FCC structures. Thus, the required restoring forces exerted on each atom to bring the NP to a single-domain equilibrium crystal become smaller. Contrary to the potential energy and the coordination number cases, there are discontinuities in the mean force–temperature curves. A smooth phenomenological relationship between the mean force and the square root of the temperature can be derived from the plotted data in both Au and Pt case. The dependence of the mean force on the NP diameter appears to weaken for larger sizes in the Au case, while it becomes stronger in the Pt case.

**Figure 8:**
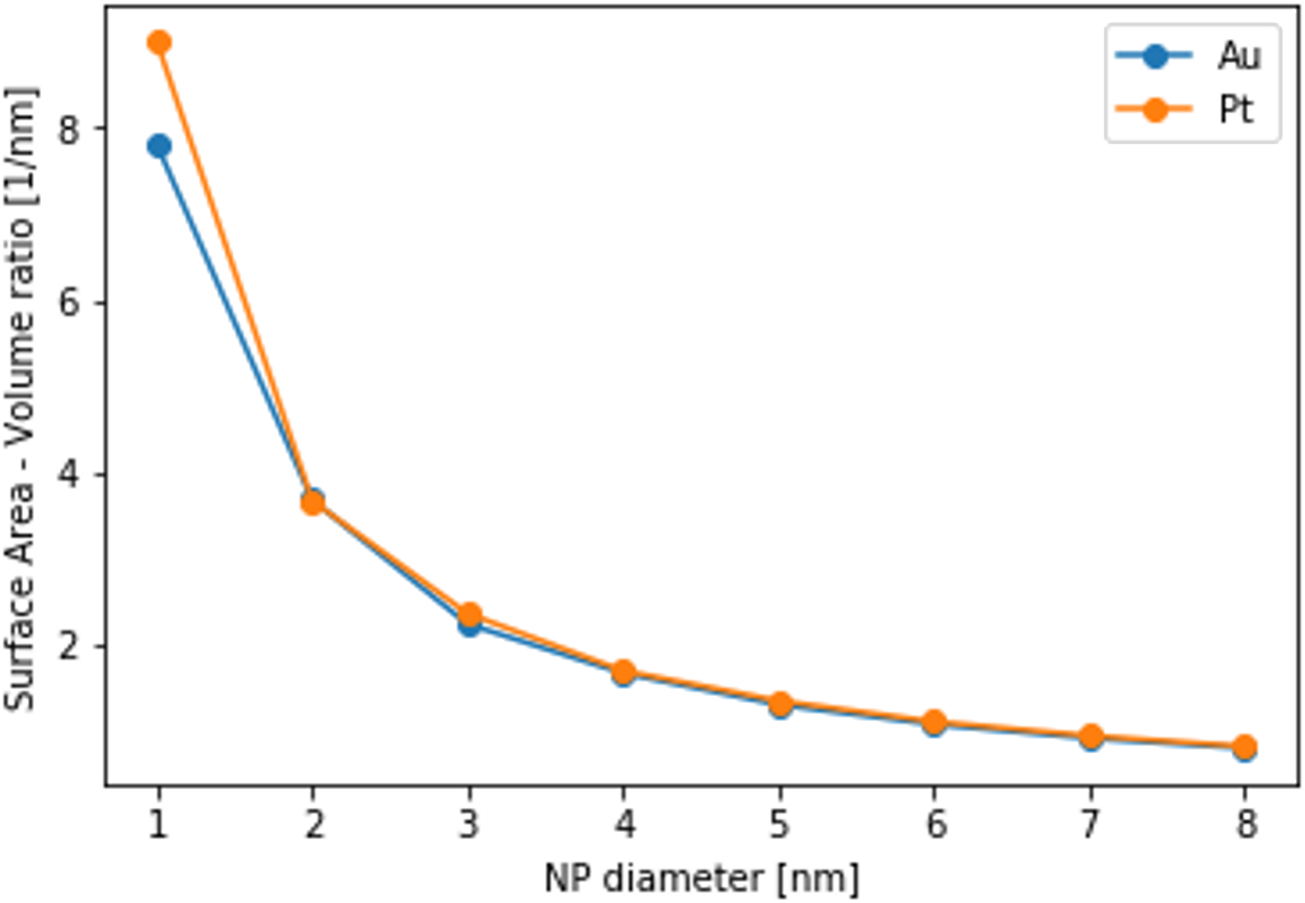
Variation of the surface area-to-volume ratio with the NP diameter for Au (blue points) and Pt (orange points) NPs. The temperature is 100 K.

One common measure of surface roughness, as well as a proxy to NP reactivity, is the surface area-to-volume ratio [83]. Its variation with the NP diameter for Au (blue line) and Pt (orange line) NPs at 100 K is shown in Figure 8. In general, it is expected to be inversely proportional to 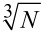 where *N* is the number of atoms in the NP. Indeed, the observed trends are in accordance with this intuitive scaling law. There are only limited differences between the Au and Pt NPs; the most notable one is for the smallest NPs with 1 nm diameter. The temperature dependence of the surface area-to-volume ratio for all NP diameters is presented in Figure S3 of Supporting Information File 1. Despite the phase transitions that the NPs undergo, the temperature dependence is weak, and a temperature increase leads to marginally higher ratios. As a conclusion, the dependence of the surface area-to-volume ratio on the NP diameter is considerably stronger than on the temperature of the NP.

**Figure 9:**
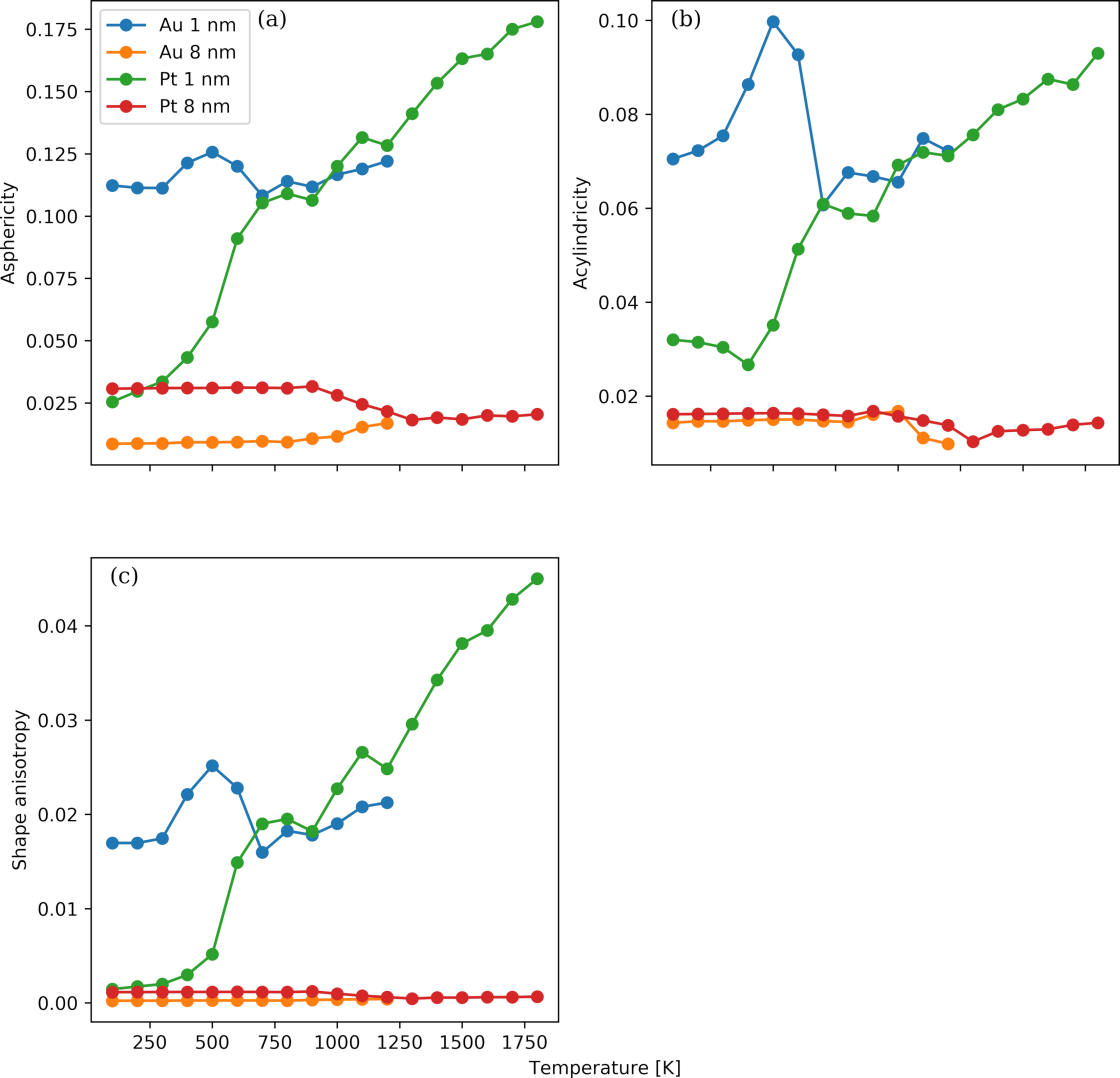
Temperature dependence of the asphericity (panel a), acylindricity (panel b), and shape anisotropy (panel c) parameters for the Au and Pt NPs with diameters of 1 and 8 nm.

The modifications in the shape of the NPs are tracked by the asphericity, acylindricity, and shape anisotropy parameters. The temperature dependence of these three parameters for the Au and Pt NPs with diameters of 1 nm (i.e., the smallest NPs) and 8 nm (i.e., the largest NPs) is shown in Figure 9. All three parameters span from zero to one. If the shape of a NP has a spherical (tetrahedral) or higher symmetry, then the parameters are equal to zero. In the case of a cylindrical symmetry, that is, the symmetry that a rigid-rod NP possesses, the acylindricity is zero, while the relative shape anisotropy is one. We observe a distinct behaviour of the small NPs compared to the large ones. In the latter case, the variation in the shape is weak, and minor changes occur only near the transition temperature identified by the Berry parameter. The actual values are close to zero, signifying a slightly deformed spherical shape, which is also confirmed by the atomistic configurations visualized in Figure S1(C,D) and Figure S2(C,D) in Supporting Information File 1. The slight increase in the asphericity parameter can be attributed to the formation of a crystallized external surface, which deviates from the curved amorphous surface structure above the transition temperature. In the case of the small Pt NPs, the parameters are proportional to the temperature and vary from values close to zero at 100 K, implying a spheroid, to values close to 0.1 or higher, implying an irregular NP form. In the case of the small Au NPs, a significant variability of the shape parameters with temperature is observed around the mean values of 0.11, 0.07, and 0.02 for, respectively, asphericity, acylindricity and relative shape anisotropy. These findings are also supported by the visualizations in Figure S1(A,B) and Figure S2(A,B). The differences between the small and the large NPs can be attributed to the higher cohesive energies of the latter.

**Figure 10:**
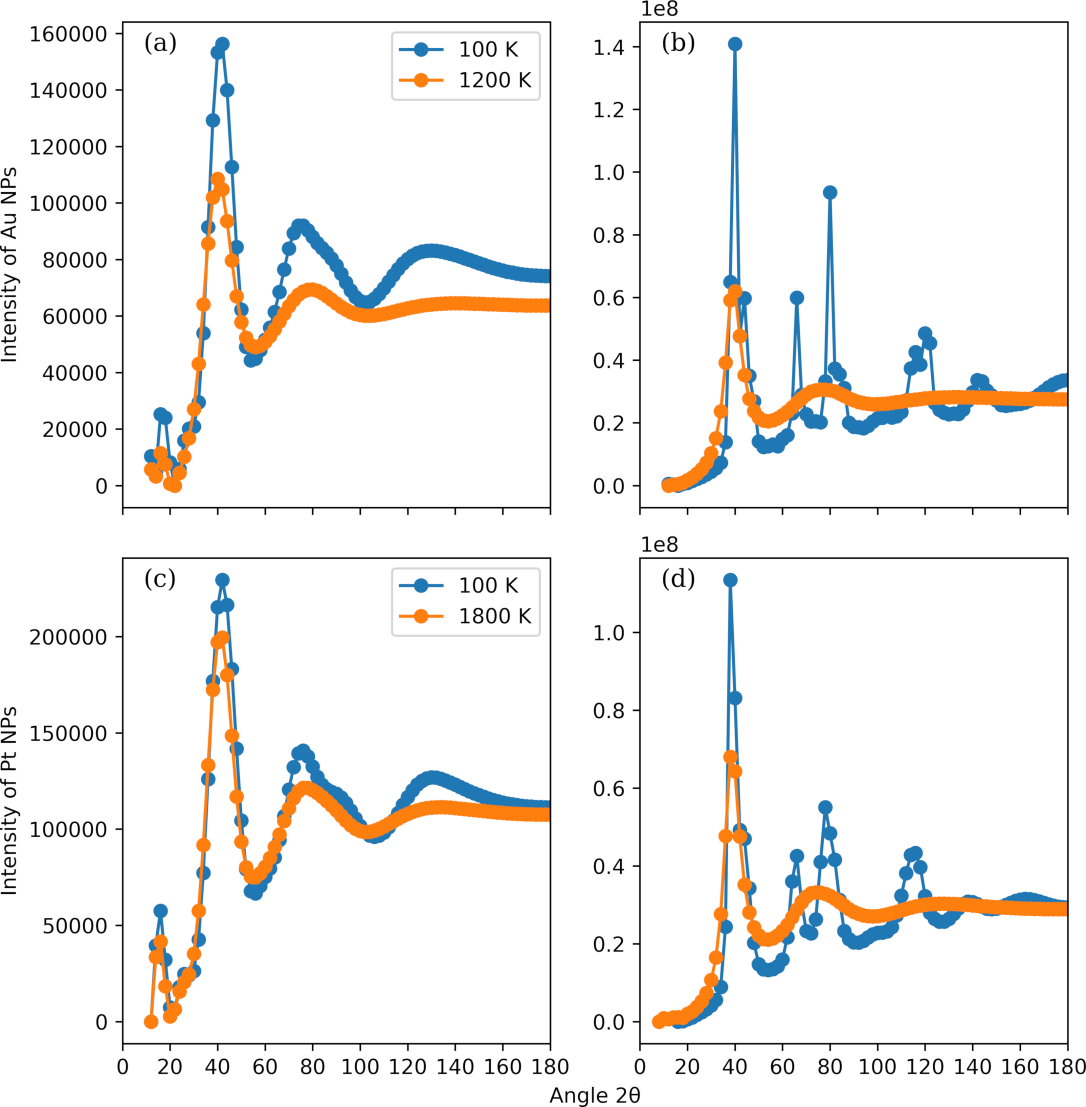
Simulated X-ray powder diffraction patterns of Au (panels a and b) and Pt (panels c and d) NPs. The NP diameters considered are 1 nm (panels a and c) and 8 nm (panels b and d). The temperatures of the Au NPs are 100 K (blue line) and 1200 K (orange line). The temperatures of the Pt NPs are 100 K (blue line) and 1800 K (orange line).

The simulated X-ray powder diffraction patterns of selected Au and Pt NPs at two temperatures are shown in Figure 10. The NP diameters are 1 nm (Figure 10a,c) and 8 nm (Figure 10b,d). The considered temperatures for the Au NPs (Figure 10a,b) are 100 K (blue line) and 1200 K (orange line), while, for the Pt NPs (Figure 10c,d), they are 100 K (blue line) and 1800 K (orange line). Similar to the number density distribution, the obtained diffraction pattern predictions at high temperature share the same characteristics regardless of the NP diameter and the chemical constitution. We observe two pairs of peaks and valleys, which are rather broad and relatively wide. For the Au NPs, the peaks are located at roughly 40° and 78°, for the Pt NPs at approximately 41° and 76°. These peaks appear also in diffraction patterns of bulk Au and Pt materials [81]. There are no persistent features in the diffraction patterns, such as peaks at multiples of characteristic length scales, and the profiles validate the notion that the NPs are amorphous. The diffraction patterns of the small NPs at 100 K are still similar to each other. They are also analogous to the patterns at high temperature, however, the peaks have become sharper, and a third distinct peak at roughly 135° is clear now. These findings support the idea that the small NPs are primarily amorphous, which can be confirmed by inspecting the temperature dependence of the Berry parameter. Much more pronounced differences are seen in the diffraction patterns of the large NPs at 100 K. These NPs have a high degree of crystallinity leading to multiple distinct peaks in their diffraction patterns. Differences between the Au and the Pt NPs are also noticeable since Au and Pt do not have the same crystallization pathways. The two peaks observed at high temperature are still present but much sharper. The new peaks in the Au (Pt) pattern are consistent with the peaks spotted at 63.032° (66.502°) and 111.486°–129.757° (115.343°–120.212°) in the pattern of a periodic bulk Au (Pt) FCC unit cell.

**Figure 11:**
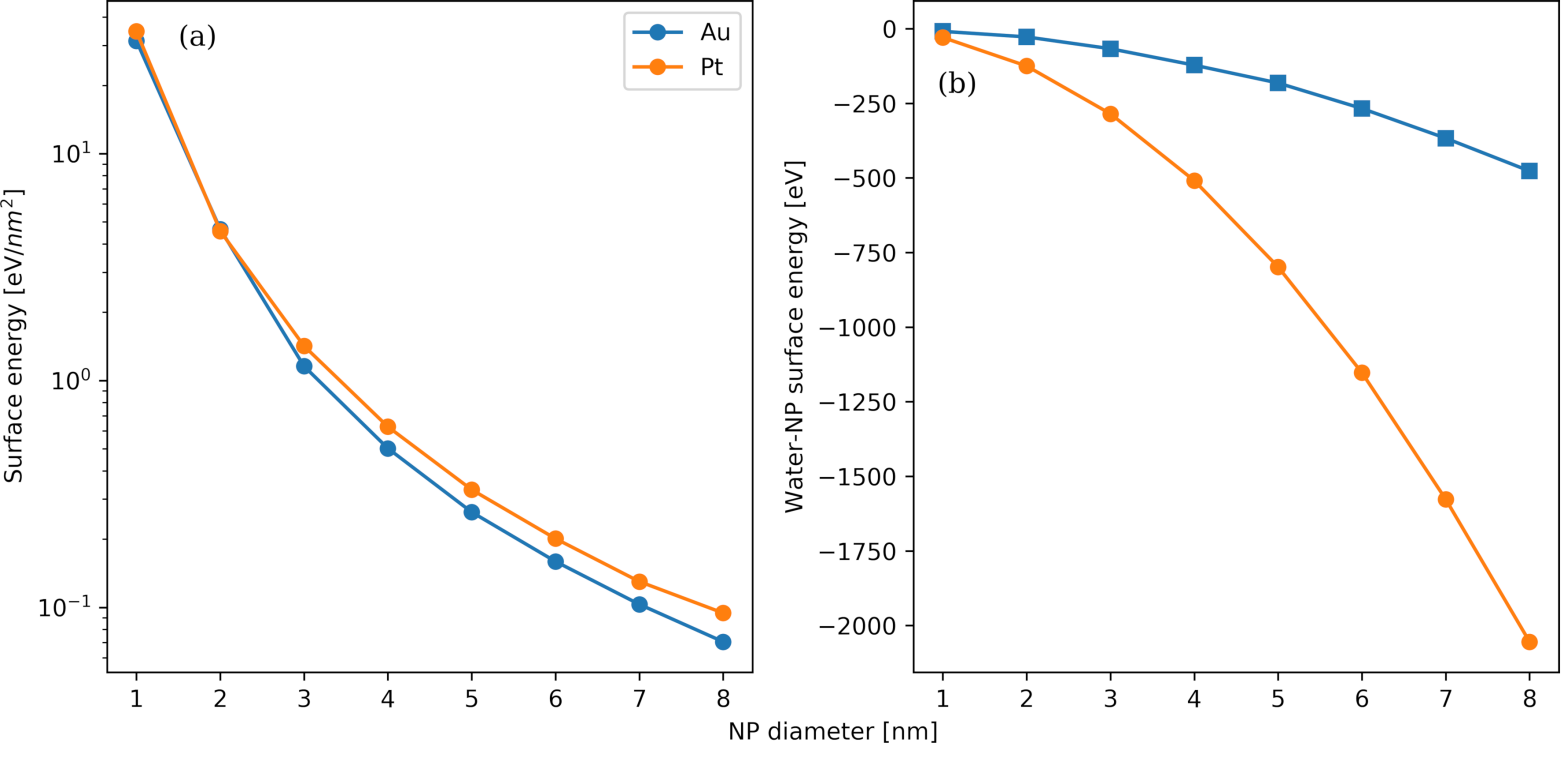
Variation of the average surface energy (panel a) and water–NP energy (panel b) with the NP diameter. The simulations have been performed at 300 K and 101.3 kPa.

In Figure 11a, the variation of the surface energy as a function of the NP diameter for Au and Pt NPs at 300 K is shown. This quantity offers an assessment of comparative stability and potential reactivity. The surface energy is determined by subtracting the potential energy of the equivalent bulk structure, for the same number of atoms, from the configuration energy of the NP. The resulting value is then divided by the surface area of the NP [84]. In general, a high value of the surface energy indicates a high potential for reactivity. We observe that the surface energy decreases with bigger NP diameters. Thus, the lesser structured amorphous spherical surfaces of the small NPs have a higher potential reactivity than the more organized crystalline multifaceted surfaces of the large NPs. This is in agreement with previous findings for Ag NPs with a similar diameter range [49]. It should be noted that the considered variations in the NP size are rather subtle and below detection for current analytical capabilities [85]. There are slight disparities between Au and Pt NPs of the same diameter, indicating that reactivity differences are expected to be limited. The surface energy of the NPs can be lowered by resorting to thiolate protection of the surface or by making use of other passivating agents. In Figure S4 of Supporting Information File 1, we provide the temperature variation of the surface energy for Au and Pt NPs with NP diameters from 1 to 8 nm. The dependence on the temperature is much less pronounced than the dependence on the NP diameter. In Figure 11b, the variation of the water–NP potential energy with the NP diameter for Au and Pt NPs at 300 K is shown. In most applications, NPs suspended in biological fluids and aqueous solutions can serve as a proxy system that is easy to control [86]. The NPs are either bare or coated with a corona, the coverage of which may fluctuate, again leaving the NP surface exposed to the solvent [87]. Thus, it is important to investigate the water–NP energetic interactions. A quadratic dependence of the water–NP potential energy on the diameter is identified; it is related to the scaling of the available NP surface for interactions with the surrounding water molecules with their diameter. Although both Au and Pt NPs interact favourably with the water solvent, the interactions are much stronger for the Pt NPs compared to the Au NPs. Therefore, the expected structural modifications and potential partial oxidation in the Pt case are going to be stronger than in the Au case. Although partial oxidation can be addressed directly via molecular simulations by means of reactive force fields [88], the size of the systems and the number of contained molecules render such an approach almost computationally unattainable.

## Conclusion

In the present simulation study, we focused on the thermal behaviour of Au and Pt NPs experiencing rapid cooling. Both Au and Pt bulk materials share the same FCC unit cell structure. The primary goal was to discern the morphological changes occurring in the NPs. An additional aim was to quantify the influence of temperature, chemical composition, and NP size on these transformations. The NPs were initially spherical, with diameters ranging from 1 to 8 nm, and melted. Because of the small size of the NPs under consideration, the structural modifications observed pose challenges for experimental techniques. The adopted approach can be readily applied to investigate other metallic and metal oxide nanomaterials.

Relatively large NPs, with a diameter greater than 3 nm, exhibit a transition temperature from a melted/amorphous state to a highly crystalline one that is nearly independent on the NP diameter. Nevertheless, it notably differs from the corresponding temperature observed for the bulk materials. The transition temperature varies significantly with size for NPs with diameters below 3 nm. Comparing Au and Pt NPs, the latter exhibit a higher degree of crystallinity under similar conditions, as revealed by the Ackland–Jones parameter and the atomic coordination number. This behaviour is attributed to the stronger cohesive forces driving the crystallization process; this is supported by inspecting the atomic potential energy and atomic forces in the NPs. Moreover, the simulated X-ray powder diffraction patterns of the nanomaterials show the formation of crystalline phases at low temperatures with the same diffraction patterns as the bulk materials. Large NPs present a multifaceted crystal surface, maintaining a nearly constant shape despite temperature fluctuations. In contrast, small NPs feature a smoother surface, while their shape varies considerably with temperature as quantified by the acylindricity and asphericity shape parameters. Indirect evidence of NP toxicity and reactivity was obtained by examining surface quantities such as the potential energy of surface atoms, the water–NP surface energy, and some descriptors that are commonly used in nano-QSAR (quantitative structure-activity relationship) models. The toxicity and reactivity are expected to be inversely proportional to the NP size but proportional to the temperature, with the former showing a more pronounced effect. Based on our results, the Pt NPs are predicted to be more reactive than the Au NPs.

## Supporting Information

The file contains four figures. The first two are visualizations of Au and Pt NPs with varying temperature and diameter. The third figure depicts the temperature dependence of surface area-to-volume ratio for Au and Pt NPs. The temperature dependence of the average surface potential energy per atom for Au and Pt NPs is shown in the last figure.

File 1 Additional figures.

## Data Availability

Additional research data is not shared.
