# Supplementary material for: Atomistic insights into the morphological dynamics of gold and platinum nanoparticles: MD simulations in vacuum and aqueous media
Source: Beilstein J Nanotechnol. 2024 Aug 7;15:995–1009. doi: 10.3762/bjnano.15.81 (PMC11318634; doi:10.3762/bjnano.15.81)
Supplement: File 1 — Additional figures. [file Beilstein_J_Nanotechnol-15-995-s001.pdf]

BEILSTEIN JOURNAL OF NANOTECHNOLOGY

Supporting Information

for

Atomistic insights into the morphological dynamics of gold and platinum nanoparticles: MD simulations in vacuum and aqueous media

Evangelos Voyiatzis, Eugenia Valsami-Jones and Antreas Afantitis

Beilstein J. Nanotechnol. **2024,** *15,* 995–1009. doi:10.3762/bjnano.15.81

Additional figures

License and Terms: This is a supporting information file under the terms of the Creative Commons Attribution License (https://creativecommons.org/licenses/by/4.0). Please note that the reuse, redistribution and reproduction in particular requires that the author(s) and source are credited and that individual graphics may be subject to special legal provisions.
The license is subject to the *Beilstein Journal of Nanotechnology* terms and conditions: (https://www.beilstein-journals.org/bjnano/terms)

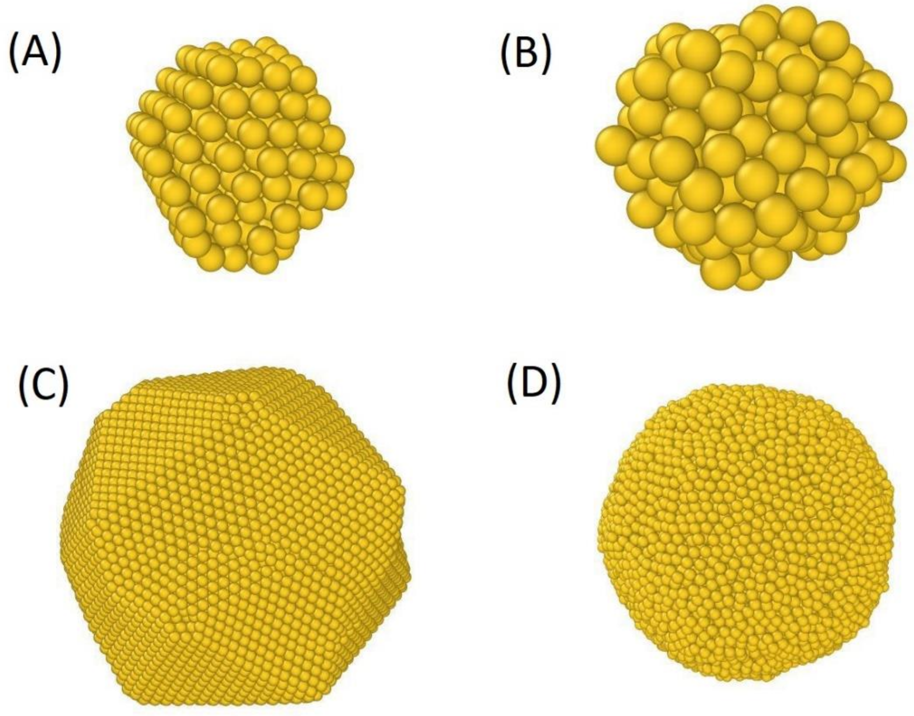

Figure S1: Visualization of Au NPs with diameters of 2 nm (panels A and B) and 8 nm (panels C and D) at 100 K (panels A and C) and 1100 K (panels B and D).

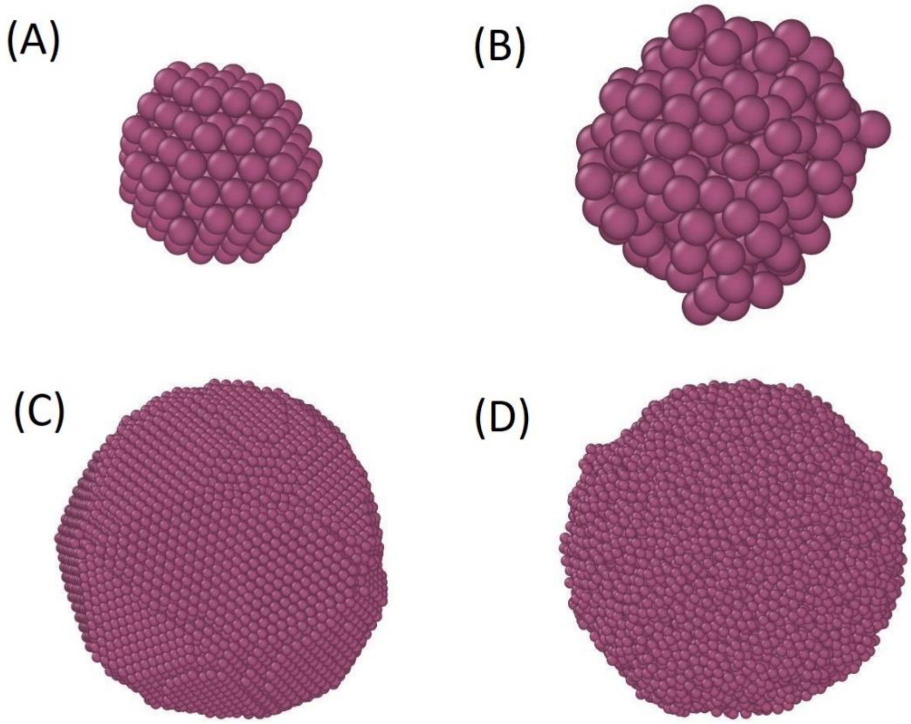

Figure S2: Visualization of Pt NPs with diameters of 2 nm (panels A and B) and 8 nm (panels C and D) at 100 K (panels A and C) and 2000 K (panels B and D).

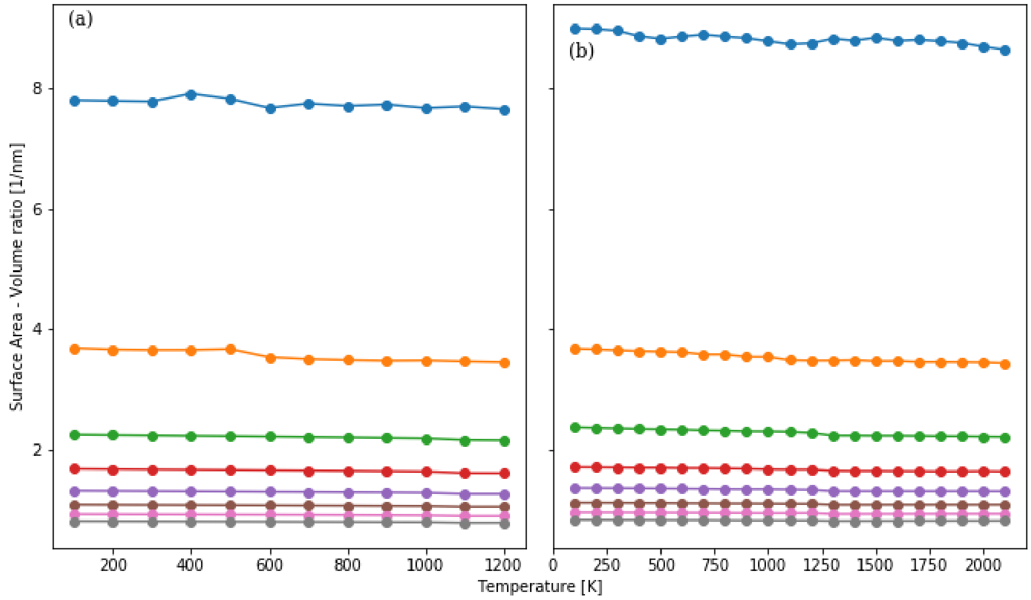

Figure S3: Temperature dependence of surface area to volume ratio for Au (panel a) and Pt (panel b) NPs. The NP diameters range from 1 nm to 8 nm. The symbols for the NP diameters are explained in Figure S4.

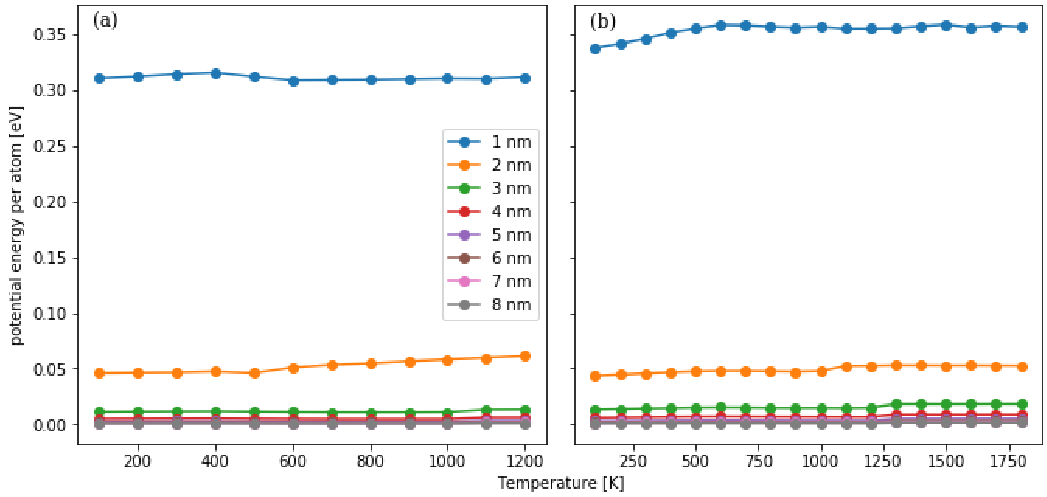

Figure S4: Temperature dependence of the average surface potential energy per atom for Au (panel a) and Pt (panel b) NPs. The NP diameters range from 1 up to 8 nm.
